# Gastrointestinal mucormycosis associated with leptospirosis: should we be concerned during major floods?

**DOI:** 10.1590/S1678-9946202567035

**Published:** 2025-06-09

**Authors:** Mariane Taborda, Juliana Possatto Fernandes Takahashi, Jessica de Brito Ferreira Nascimento, Julia Ferreira Mari, Vítor Falcão de Oliveira, Adriana Satie Gonçalves Kono Magri, Ana Catharina de Seixas Santos Nastri, Marcello Mihailenko Chaves Magri

**Affiliations:** 1 Universidade de São Paulo, Faculdade de Medicina, Hospital das Clínicas, Departamento de Moléstias Infecciosas e Parasitárias, São Paulo, São Paulo, Brazil; 2 Instituto Adolfo Lutz, Unidade de Patologia Quantitativa, São Paulo, São Paulo, Brazil

**Keywords:** Gastrointestinal mucormycosis, Mucormycosis, Leptospirosis, Enterorrhagia

## Abstract

Gastrointestinal mucormycosis (GIM) and leptospirosis are two severe diseases associated with high morbidity and mortality rates. The coexistence of these two conditions has not yet been reported in the literature. This study presents a case involving this rare association. A 49-year-old man from Sao Paulo, Brazil, was hospitalized with acute abdominal pain, low blood pressure, and jaundice. He had a history of contact with floodwater and ingestion of contaminated water, was hospitalized with acute abdominal pain, low blood pressure, and jaundice. Upon ICU admission, he developed jaundice, acute renal failure requiring hemodialysis, and alveolar hemorrhage necessitating intubation. Leptospirosis was confirmed by serological tests and treated with ceftriaxone for 14 days. Two weeks later, he developed severe enterorrhagia, requiring a massive transfusion and a total colectomy with terminal ileostomy. Histopathology revealed necrotizing granulomatous inflammation with hyphae indicative of mucormycosis. He was treated with amphotericin B for 7 weeks, followed by posaconazole. Abdominal CT scans over the next five months showed complete clinical and radiological improvement. The association between mucormycosis and leptospirosis has not been previously documented, highlighting the diagnostic challenges and the critical importance of early detection. Successful management in this case required timely surgical intervention combined with prolonged antifungal therapy.

## INTRODUCTION

Gastrointestinal mucormycosis (GIM) is a rare fungal infection associated with high mortality rates of approximately 40% in adults and 70% in neonates^1^. It may manifest as a generalized or localized infection, most commonly affecting the stomach, colon, or small intestine. Its incubation period is uncertain and the disease presentation is often nonspecific, making diagnosis challenging. Risk factors in non-classically immunosuppressed patients include hemodialysis, broad-spectrum antibiotic use, and malnutrition^1–3^.

Leptospirosis is a bacterial zoonosis disease caused by *Leptospira* species, typically associated with heavy rainfall and transmitted through contact with contaminated urine or environments. The incubation period can range from 1 to 30 days, although it typically occurs between 7 and 14 days after exposure^4,5^. Its severe form, known as Weil's syndrome, is characterized by liver, kidney, and pulmonary failure^4^. *Leptospira* invade capillaries by adhering to VE-cadherin, disrupting endothelial cells, and causing edema and hemorrhage^5^. Coinfections with leptospirosis are rare but should be considered in regions where clinical presentations overlap with diseases like dengue, Zika, and malaria^6,7^.

This study presents a rare case of GIM associated with leptospirosis, highlighting the importance of detecting such conditions in the context of natural disasters, particularly flooding events. The research complied with the Declaration of Helsinki and was approved by the Research Ethics Committee of the Faculdade de Medicina da Universidade de Sao Paulo, Brazil (protocol N° 69080023.2.0000.0068, approved at January 19, 2023).

## CASE REPORT

A 49-year-old man living in Sao Paulo, Brazil, and working as a marble and tile setter in the construction industry, was admitted to the hospital with acute, diffuse abdominal pain, arterial hypotension, and jaundice. The patient reported exposure to floodwater and ingestion of contaminated water 10 days prior to symptom onset. On admission, an abdominal computed tomography (CT) scan revealed pneumoperitoneum near the gallbladder and bile ducts. An exploratory laparotomy with cholecystectomy was performed, but no gastroduodenal perforation was observed. The patient remained in the intensive care unit (ICU), where he developed jaundice, acute renal failure requiring hemodialysis, and alveolar hemorrhage necessitating orotracheal intubation. Laboratory investigation confirmed leptospirosis by two consecutive serological tests: ELISA-IgM and a microscopic agglutination test for the serovar Cynopteri, with a titer of 1/800. He was treated with ceftriaxone for 14 days, leading to clinical and laboratory improvement.

After two weeks hospitalized, the patient experienced multiple episodes of severe enterorrhagia and required a massive transfusion of 10 units of red blood cells. Due to ongoing bleeding, a total colectomy with terminal ileostomy was performed, and the resected samples were sent for histopathological examination. The patient showed clinical improvement and was discharged for outpatient follow-up.

**Figure 1:**
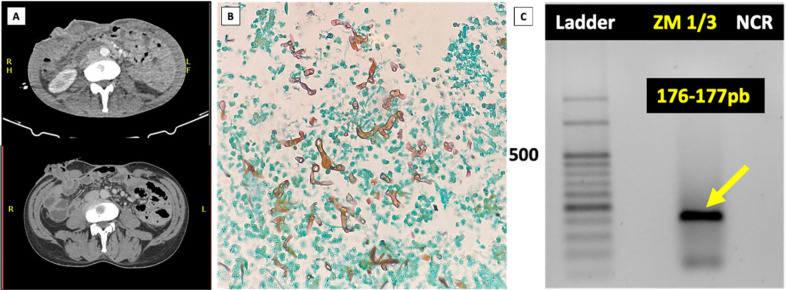
(A) Radiographic signs of Gastrointestinal mucormycosis. Signs of total colectomy. Ileostomy in the right flank without particularities. Rectal stump filled with hyperattenuating content, nonspecific. Diffuse thickening of the small bowel loops with slight distension, without a clearly defined obstructive factor, associated with diffuse densification of the peritoneal planes and folds. The findings are nonspecific, suggesting congestion or inflammatory changes. Small amount of free abdominal fluid. Partial thrombosis of the right external iliac vein, now recanalized. Dehiscence of soft tissues in the midline abdominal suture without intraperitoneal exposure. Diffuse textural changes of the skeletal framework; (B) Hyphal morphology in Gastrointestinal mucormycosis. Typical hyphal morphology in mucormycosis lesions (Grocott stain). Mucorales hyphae are ribbon-like, pauci-septate, and branch irregularly; (C) Electrophoresis was performed using a Gel Doc imaging system (Bio-Rad) to visualize the specific amplicon sizes, revealing a fragment generated with the ZM1/3 primers (176-177 pb).

One month later, he returned to the hospital with a surgical wound infection. An abdominal CT scan showed diffuse parietal thickening of the small bowel loops, mild distension without obstructive factors, and diffuse densification of the peritoneal layers (Figure 1A). At this time, histopathology from the first surgery revealed a necrotizing granulomatous inflammatory process, a transmural ulcer with hyphae suggestive of mucormycosis (Figure 1B), fibrino-leukocytic peritonitis, acute purulent periappendicitis, reactive lymphoid hyperplasia, and chronic epiploitis with suppuration foci. Mucormycosis was subsequently confirmed by PCR on the paraffin-embedded tissue sample from the total colectomy (Figure 1C). The patient was started on liposomal amphotericin B at a 5 mg/kg/day dosage for 7 weeks, followed by oral posaconazole suspension at 400 mg twice per day. Follow-up abdominal CT scans showed complete clinical and radiological resolution after five months of posaconazole therapy.

## MATERIALS AND METHODS

### Proven mucormycosis (EORTC/MSG 2020 criteria)

Mucormycosis was confirmed based on the 2020 EORTC/MSG criteria for proven invasive fungal disease, which require histopathological evidence of hyphae in a tissue biopsy, accompanied by signs of associated tissue damage^8^.

### DNA extraction protocol

Genomic DNA was extracted from fresh or formalin-fixed paraffin-embedded (FFPE) tissue samples using a magnetic bead-based extraction method (Zymo Research, Irvine, CA, USA). Approximately 200 μL of DNA/RNA Shield (1×) was added to each sample, followed by vigorous vortexing to homogenize the material. For enzymatic digestion, 15 μL of Proteinase K (20 mg/mL) was added and mixed thoroughly by pipetting. Subsequently, 400 μL of Viral DNA/RNA Buffer was added, the samples were vortexed for 5 s, and briefly centrifuged. Samples were then incubated at 56 °C in a thermomixer set at 650 rpm (or in a static heat block), until complete lysis of the tissue was achieved—typically after 3 h or overnight for FFPE or fresh tissues, and 30 min for other sample types. To inactivate residual proteinase activity, samples were incubated at 85 °C for 10 min and centrifuged to collect condensation. Next, 30 μL of MagBinding Beads was added to each lysate. The mixture was vortexed and incubated at 25 °C for 10 min with agitation at 1,300 rpm. After brief centrifugation, the tubes were transferred to a magnetic rack until the beads were visibly pelleted. The supernatant was carefully removed using a 1000 μL pipette without disturbing the beads. The tubes were then removed from the magnetic rack, and 500 μL of MagBead DNA/RNA Wash 1 was added. After vortexing for 10 s and centrifugation, the samples were returned to the magnetic rack, and the supernatant was discarded. This washing process was repeated using 500 μL of MagBead DNA/RNA Wash 2, followed by two additional washes with 250 μL of 95–100% molecular biology grade ethanol. After each wash, samples were vortexed, centrifuged, and cleared of supernatant following magnetic separation. After the final ethanol wash, samples were centrifuged at 13,000 rpm for 2 min, and residual ethanol was removed. The tubes were then incubated at 37 °C for at least 1 h (with lids closed or partially sealed using parafilm) until the beads appeared completely dry. DNA was eluted by adding 30–60 μL of DNase/RNase-Free Water, followed by vortexing and centrifugation. The eluate was separated from the beads using a magnetic rack and transferred to a clean, labeled microcentrifuge tube. Purified DNA samples were stored at −80 °C until further use.

### Mucorales semi-nested polymerase chain reaction

Mucorales semi-nested polymerase chain reaction (cPCR) was conducted using 13.0 μL of GoTaq^®^ (Promega, Madison, WI, USA) DNA Polymerase Master Mix, which contains 2X Green GoTaq^®^ Reaction Buffer (pH 8.5), 400 μM dATP, 400 μM dGTP, 400 μM dCTP, 400 μM dTTP, and 3 mM MgCl_2_. The first amplification step included 1 μL of the forward primer ZM1 (20 μM) (5’-ATT ACC ATG AGC AAA TCA GA-3’), 1 μL of the reverse primer ZM2 (20 μM) (5’-TCC GTC AAT TCC TTT AAG TTT C-3’), and 10 μL of extracted DNA, yielding a final reaction volume of 25 μL. For the second amplification step, 13.0 μL of GoTaq^®^ Master Mix was used, along with 1 μL of the forward primer ZM1 (20 μM) and 1 μL of the reverse primer ZM3 (20 μM) (5’-CAA TCC AAG AAT TTC ACC TCT AG-3’)^9–11^. A total of 10 μL of the first-round PCR product was used as template, resulting in a final reaction volume of 25 μL. Thermal cycling conditions consisted of an initial denaturation at 94 °C for 5 min, followed by 35 cycles of denaturation at 94 °C for 30 s, annealing at 50 °C for 30 s, and extension at 72 °C for 1 min, with a final extension step at 72 °C for 5 min^9^. Negative controls were employed at all stages to ensure absence of contamination. Electrophoresis was performed using a Gel Doc imaging system (Bio-Rad, Hercules, CA, USA) to visualize the specific amplicon size. The products of the semi-nested reaction using primers ZM1/ZM2 and ZM1/ZM3 are 407 to 408 bp and 175 to 177 bp in length, respectively, depending on the species^9^.

## DISCUSSION

Leptospirosis causes thousands of deaths each year worldwide and is a growing public health problem exacerbated by urbanization, global warming, climate change, and increasing poverty^11^. In Brazil, seasonal floods during the summer are a major contributing factor to the high disease burden, although the actual number of infections is likely underestimated^12^. Despite the high global incidence of leptospirosis, to our knowledge this is the first reported case of GIM associated with Weil's syndrome.

GIM can result from various sources, including contaminated hospital materials and prostheses, infected wooden tongue depressors, nasogastric tubes, contaminated intravenous fluids, ostomy bags, catheters, intravascular devices, surgical procedures, and peritoneal dialysis. It has also been linked to bacterial infections, such as typhoid fever-related ulcerations, and protozoan infections, like colitis caused by *Entamoeba histolytica* ^1,2,13^. GIM has recently been reported in association with COVID-19 in countries like India and Brazil^14,15^. Contaminated water and food, including fermented milk, dry bread products, and alcoholic beverages made from infected corn, have also been associated with *Mucorales* infections^16^.

Recent studies have reported GIM in patients with classic predisposing conditions, including diabetes mellitus, immunosuppression due to hematologic malignancy, stem cell or solid organ transplantation, steroid therapy, iron overload, chemotherapy, malnutrition, and chronic alcohol abuse^1,2,17^. In neonates, the most commonly identified risk factors include prematurity, low birth weight, gastrointestinal interventions, endotracheal intubation, systemic inflammatory response syndrome, shock, metabolic acidosis, malnutrition, and ICU admission^18^. A systematic review by Didehdar *et al*.^2^ analyzing GIM cases from 2015 to 2021 reported that 11% of patients were immunocompetent and had no identifiable underlying disease. Our patient, a construction worker handling marble and tile materials, had no known immunosuppressive conditions only a history of contact with floodwater and potentially contaminated drinking water. In his profession, he was likely exposed to rat urine contaminating water puddles, materials, and work equipment. During his hospital stay, notable invasive procedures included the use of catheters, tubes, and dialysis, among others.

A review of 200 GIM cases from 1947 to 2017 showed that most occurred in Asia (50.6%) compared with South America (n = 4; 2.3%). Of 176 cases with available age data, 89 (50.6%) were adults, and 87 (49.4%) were children, including 61 (70.1%) neonates^1^. Another systematic review of 87 GIM cases from 2015 to 2021 identified only three cases in Brazil. In this cohort, 46% of patients were female and 54% were male, with a mean age of 46 years (range: 9–84 years). Conversely, among neonatal patients, 78% were male and 22% were female. Mortality rate was 70% in neonates and 44% in older patients^2^.

GIM most commonly involves the stomach (55%), followed by the small intestine (40%) and colon (34%), consistent with our case, which progressed from Weil's syndrome and ultimately required emergency surgery, resulting in a total colectomy due to mucormycosis. Mucorales have also been reported to infect the liver and esophagus, appendix, spleen, and pancreas^1,2^. Kaur *et al*.^1^ observed that the stomach was the organ most commonly affected in adults, whereas the large intestine and small intestine were significantly involved in children.

The most frequently reported GIM manifestations include abdominal pain (50%), fever (37%), and gastrointestinal perforation (27%)^1,2^. GIM clinical manifestations are often nonspecific and can mimic other conditions. Hence, differential diagnoses for GIM include diverticulitis, cytomegalovirus infection, pseudomembranous colitis, inflammatory bowel disease, lymphoma, appendicitis, cholecystitis, Ogilvie's syndrome, cholangitis, and complications from radiotherapy or chemotherapy^1,2,19^. In our case, the patient's symptoms were consistent with both leptospirosis and mucormycosis; however, mucormycosis diagnosis was only confirmed after the total colectomy performed due to severe enterorrhagia.

Imaging findings on CT scan in cases of colonic involvement may include diffuse wall thickening, lack of bowel wall visualization, pneumatosis, adjacent air or fluid collections, and areas of contrast extravasation or pooling within the lumen. These findings can also extend to involve the small bowel and appendix, presenting as non-enhancing areas, necrosis, or periappendiceal collections^19^. In our patient, the abdominal CT findings—despite being nonspecific and common in various abdominal pathologies—were consistent with descriptions in the literature, even post-surgery. This overlap complicates diagnosis. Thus, maintaining a high index of suspicion for GIM is crucial, particularly in critically ill patients who present with abdominal pain, distension, enterorrhagia, and signs of perforation.

Histopathology is essential for diagnosing GIM. Confirming infection requires the observation of characteristic hyphae within tissue samples, which are typically hyaline (non-pigmented) and show tissue invasion when stained with hematoxylin and eosin (HE), periodic acid-Schiff (PAS), or Grocott-Gomori methenamine silver stains. Histologically, the hyphae are usually broad, ribbon-like, and irregular, measuring 6 to 25 μm or more in width, and are coenocytic (non-septate) or pauciseptate with right-angle branching. Immunohistochemistry using commercially available monoclonal antibodies can assist in confirming the diagnosis in uncertain cases. Additionally, PCR techniques on fresh, paraffin-embedded, or formalin-fixed tissue have shown high specificity for identifying Mucorales^9,10,19^.

The most commonly used antifungals in clinical practice for mucormycosis are amphotericin B, posaconazole, and isavuconazole. Currently, no randomized controlled trials have compared different antifungal agents or formulations of amphotericin B in humans. Mucormycosis treatment should, whenever possible, include a combination of aggressive surgical debridement with a clear safety margin, antifungal therapy, and reversal or control of predisposing factors, with particular emphasis on glycemic control. International guidelines recommend liposomal amphotericin B at doses of 5 mg/kg or higher for 2 to 6 weeks, followed by sequential therapy with isavuconazole or posaconazole for an extended period^19^.

Regarding leptospirosis, Oliveira *et al*.^20^ reported the detection of antibodies against *Leptospira kirschneri* serogroup Cynopteri among residents of low-income urban communities in Salvador, Brazil, suggesting a distinct epidemiological pattern compared with *L. interrogans* serogroup Icterohaemorrhagiae. Although the overall seroprevalence of Cynopteri was low (1.6%), significant associations were found with increasing age, residence in houses with unplastered walls, and the presence of cats in the peridomestic environment. These findings indicate a potentially different transmission cycle, possibly involving alternative reservoirs that remain unidentified in the urban context. Seropositivity to Cynopteri, particularly in areas lacking classical risk factors like sewage exposure or occupational hazards, raises the hypothesis that other animals, such as cats or even bats, the original host species from which this serovar was first isolated, may play a role in its urban maintenance. The identification of this serogroup underscores the need for further investigation into non-traditional urban reservoirs and reinforces the importance of considering the ecological diversity of circulating Leptospira serogroups, even when therapeutic approaches remain unaffected by the infecting serovar.

## CONCLUSION

In conclusion, GIM is a rarely reported disease in Brazil but is associated with high morbidity and mortality rates. Effective management requires addressing underlying conditions, using multiple diagnostic techniques, and implementing appropriate antifungal therapy in combination with surgery. The association between mucormycosis and leptospirosis has not been previously described, underscoring the need for further research to better understand this interaction. We recommend considering GIM in adult patients presenting with gastrointestinal bleeding, abdominal distension, fever, vomiting, or an abdominal mass, particularly in the presence of predisposing risk factors for mucormycosis. Increased awareness of GIM can enhance clinical suspicion, potentially facilitating earlier diagnosis and prompt treatment.
